# Enhanced Schwann cell differentiation of skin‐derived neural crest‐like stem cells through the synergistic action of SOX10 and immobilized NRG1 signaling

**DOI:** 10.1002/btm2.70041

**Published:** 2025-08-20

**Authors:** Ashis Kumar Podder, Pihu Mehrotra, Pedro Lei, Stelios T. Andreadis

**Affiliations:** ^1^ Department of Chemical and Biological Engineering University at Buffalo, The State University of New York (SUNY) Buffalo New York USA; ^2^ Department of Biomedical Engineering University at Buffalo, SUNY Buffalo New York USA; ^3^ Center of Excellence in Bioinformatics and Life Sciences University at Buffalo, SUNY Buffalo New York USA; ^4^ Center of Cell, Gene and Tissue Engineering University at Buffalo, SUNY Buffalo New York USA; ^5^ Present address: Department of Chemical and Biological Engineering Princeton University Princeton New Jersey USA

**Keywords:** neural crest cells, Schwann differentiation, SOX10, NRG1, cell therapy, peripheral nerve repair, autologous Schwann cells

## Abstract

Human skin‐derived neural crest (NC)‐like stem cells present a highly accessible, autologous source of multipotent cells, with the potential to differentiate into a variety of cell types, including Schwann cells (SCs). However, these cells quickly lose their stem‐like characteristics in vitro and eventually limit their ability to form functional SCs. To overcome this, we investigated SOX10 upregulation, the key regulator of NC formation and multipotency, using both small chemical (Forskolin and RepSox) treatment and genetic modification. Remarkably, SOX10 upregulation highly increased SC gene expression instead of NC markers, though Forskolin‐RepSox also triggered melanocytic and smooth muscle gene markers alongside reduced NC genes. In contrast, genetic SOX10 upregulation enhanced both SOX10 and NC gene expression without inducing alternative lineages. Continuous SOX10 expression was necessary for increased SC protein markers, and differentiating SOX10‐overexpressing cells on immobilized NRG1 further enhanced SC markers and induced a distinct, elongated morphology typical for myelinating SCs. Therefore, this study introduces a rapid, efficient method to derive SC‐like cells from the skin‐derived NCs, highlighting their potential in regenerative medicine for cell therapy and disease modeling applications.

## Graphical abstract

This study establishes an efficient strategy to generate autologous Schwann‐like cells from KC‐NCs via sustained SOX10 overexpression. Differentiation on immobilized NRG1 enhances mature SC markers and induces bipolar morphology, supporting their therapeutic potential for peripheral nerve repair, disease modeling, and regenerative medicine applications.

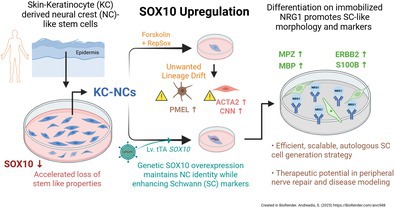

**Translational Impact Statement**

This study presents a robust strategy to generate autologous Schwann cell (SC)‐like cells from human skin‐derived keratinocyte‐induced neural crest (KC‐NC) cells. Sustained SOX10 upregulation significantly enhances SC marker expression and differentiation while maintaining NC‐like characteristics in undifferentiated cells. Differentiation on immobilized NRG1 further promotes mature SC markers and induces bipolar morphology, typical of early‐mature SCs transitioning toward a myelinating phenotype. This efficient and scalable approach of obtaining SC‐like cells highlights their therapeutic potential in peripheral nerve repair, disease modeling, and cell‐based regenerative medicine applications.

## 1 INTRODUCTION

The neural crest (NC) is a unique, multipotent and migratory population of stem cells critical for vertebrate embryonic development. NCs differentiate into diverse cell lineages such as cranio‐facial bone and cartilage, pigment cells, neuronal, and glial cells of the peripheral and enteric nervous system.^1^ Unlike typical stem cell populations, NC cells have a limited capacity for both self‐renewal and multipotency.^2^ Among their derivatives, Schwann cell precursors are one of the most multipotent types and can differentiate into neurons, melanocytes, and Schwann cells (SCs).^3^, ^4^ SCs play an essential role in myelinating neurons of the peripheral nervous system (PNS) and facilitating nerve repair after injury by clearing myelin debris and supporting remyelination of the injured axons.^3^, ^4^

Peripheral nerve injuries (PNI) affect approximately 0.18% of the population annually (an estimated 18 individuals per 1,000,000),^5^ often due to peripheral nerve injuries caused by trauma and medical conditions, necessitating generation of functional SCs for therapeutic applications. Autologous, or patient‐specific, NC‐like stem cells from accessible postnatal (human) tissues such as hair follicles,^6^ dental pulp,^7^ bone marrow,^8^ and skin‐derived mesenchymal stem cells (MSCs)^9^ – have shown potential for generating SC‐like cells for regenerative applications. In our lab, we have isolated NC‐like stem cells from neonatal foreskin‐^10^ and adult skin‐^11^ derived keratinocytes (KCs), referred to as KC‐NC cells. Given the skin's abundance and easy accessibility, these KC‐NC cells hold promise for regenerative medicine applications for PNS myelopathies.

However, like their embryonic counterparts, KC‐NCs quickly lose their multipotency in culture, as evidenced by a decline in expression of key NC specifier genes and stemness‐associated genes within 7 days post‐induction of the skin KCs to KC‐NCs.^12^ This presents a significant challenge for expanding and differentiating the KC‐NCs for potential cell therapy applications. Of all the genes associated with NC identity, *Sox10* is widely recognized as a master regulator of NC development, multipotency, cell fate determination, and subsequent differentiation.^13^, ^14^ Sox10 also supports all aspects of SC development,^15^, ^16^ through regulators such as Oct6 and Krox20,^17^ facilitating transactivation of S100B^18^ and expression of mature SC proteins such as MPZ,^19^ MBP, PMP22, and Connexin 32.^20^

Sox10 plays a critical role in both stem cell maintenance and commitment to the SC lineage during embryonic development.^16^ However, it remains unclear whether Sox10 performs a similar function in stem cells derived from adult tissues. In addition, sustained signaling from immobilized neuregulin 1 (iNRG1), an important ligand mediating the interaction of SC with neuronal axons immobilized, was shown to promote SC maturation and function as evidenced by alignment with rat DRG neurons in vitro and TUJ1^+^ neuronal axons in vivo (Tseropoulos, Mehrotra et al. 2024). In this study, we hypothesized that upregulating SOX10 might enhance KC‐NC differentiation toward SC fate. Furthermore, the combination of SOX10 with sustained iNRG1 signaling might synergistically enhance KC‐NC differentiation toward mature Schwann‐like cells, suitable for regenerative medicine applications.

## 2 RESULTS

### 2.1 Small molecule‐mediated KC‐NC induction upregulated SOX10 and triggered morphological changes

**FIGURE 1:**
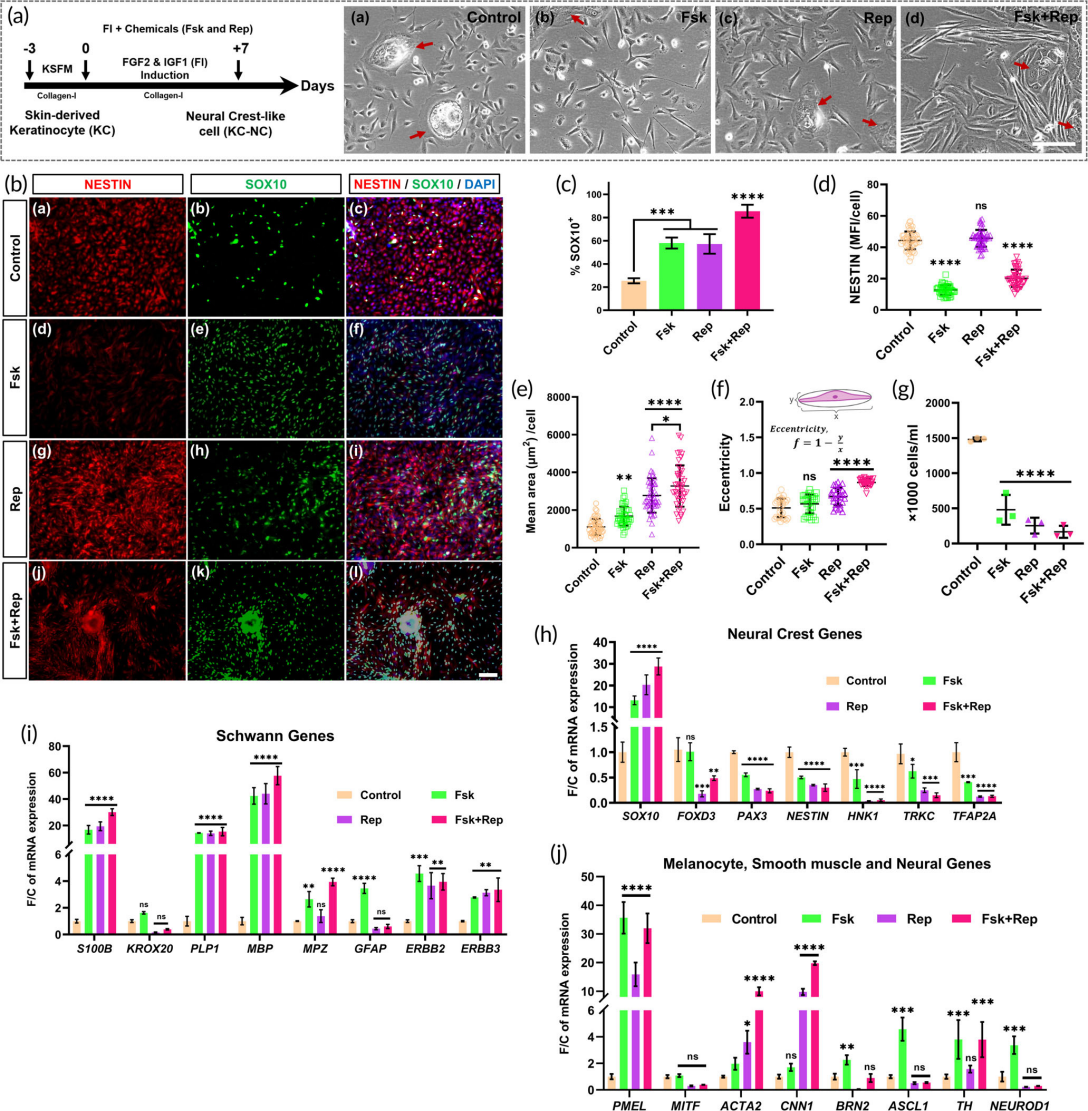
Chemically induced upregulation of *SOX10* enhances Schwann cell (SC) specific gene expression in neural crest (NC)‐like cells. (A) Schematic representation of FGF2 and IGF1 (FI) mediated reprogramming for generating NC‐like cells (KC‐NCs) from neonatal skin‐derived keratinocytes (KC) and representative phase images of the KC‐NCs following the 7‐day induction of KCs with (a) Control, (b) Fsk, (c) Rep, and (d) Fsk + Rep. Red arrows indicate the KC colonies from which the KC‐NCs originate. Scale bar: 200 μm. (B) Representative immunofluorescence images for cytoplasmic NESTIN (red), nuclear SOX10 (green), and nuclear DAPI (blue) following (a–c) no treatment (Control) or treatment with (d–f) Forskolin (Fsk), (g–i) RepSox (Rep), and (j–l) their combination (Fsk + Rep). Scale bar: 200 μm. (C, D) Quantification of the percentage (%) of SOX10^+^ cells over the total number of KC‐NCs and mean fluorescence intensity (MFI) of NESTIN (cytoplasmic) in control (FI) and Fsk + Rep (FI) treated cells evaluated after 7 days. (E) Quantification of average cell area and (F) cell elongation measured as eccentricity, *f*, which is calculated from the measurements of major (x) and minor (y) axis length of a cell using ImageJ. An *f* value approaching 1.0 indicates a highly elongated, bipolar morphology, while values closer to 0.0 suggest a circular shape. (G) Measurement of cell proliferation quantified as the number of cells obtained after differential trypsinization of the KC‐NCs from KC colonies following the 7‐day induction period. (H–J) Quantification of gene expression via quantitative RT‐PCR for key genes specific to (H) NC cells, (I) SCs, (J) melanocytes, smooth muscle cells, and neurons. Data are presented as fold change (F/C) in mRNA level normalized to control (day 7, untreated KC‐NCs) and internally normalized to *RPL32* cycle number. All data in bar graphs are presented as mean ± SD. All statistical tests were performed using one‐way analysis of variance (ANOVA) with Tukey's multiple comparison test; *n* = 3 different KC donors; ns: *p* ≥ 0.05, *: *p* < 0.05, **: *p* < 0.005, ***p: <0.0005, ****: *p* < 0.0001.

Previously, we reported that key NC specifier genes (*SOX10, PAX7, HNK‐1, MYCN*) and pluripotency genes (*SOX2, KLF4, NANOG*) were significantly downregulated during the first 7 days of KC‐NC induction, indicating a swift loss of KC‐NC multipotency and self‐renewal capacity.^12^ To counteract this loss and maintain NC‐like identity, we evaluated combinations of three small molecules known for epigenetic regulation and signaling modulation in NC cells^21^: Forskolin (Fsk) known for activating cAMP (cyclic adenosine monophosphate), valproic acid (VPA), a histone deacetylase (HDAC) inhibitor, RepSox (Rep), an ALK‐5 receptor inhibitor of TGF‐β signaling (Figures 1A and S1A).

In our initial screening, we assessed SOX10 and NESTIN expression (Figure S1B,C) in the KC‐NCs at two time points, day 7 and day 10, post‐treatment. Compared to Control, treatments with Fsk, Rep, and their combination (Figure 1B,C) significantly increased the percentage of SOX10^+^ cells (Control: 25.5 ± 2.2%, Fsk: 58.0 ± 4.7%, Rep: 57.3 ± 8.5%, Fsk + Rep: 85.5 ± 5.6%, *p* < 0.0001, Dunnett's post hoc test) on day 7. In contrast, VPA (10.2 ± 2.0) showed minimal SOX10 expression on day 7 (Figure S1B(h),D). However, by day 10, SOX10 expression declined for most conditions (Figure S1C,D), (Control: 8.1 ± 1.4%, *p* < 0.0001; Fsk: 43.8 ± 1.2%, *p* = 0.0006; Rep: 50.3 ± 2.5%, *p* = 0.2211; Fsk + Rep: 73.2 ± 2.9%, *p* = 0.0034, Dunnett's post hoc test), indicating partial maintenance of NC‐like identity. The Fsk + VPA combination failed to induce NC‐like cells by day 7, while extended treatment resulted in cell death (Figure S1B,C(p–r)).

NESTIN expression showed no significant changes between Control, VPA, Rep, and VPA + Rep treatments on day 7 (Control: 44.3 ± 5.7; VPA: 35.6 ± 3.0, *p* = 0.0205; Rep: 45.7 ± 5.4, *p* = 0.3777; VPA + Rep: 47.9 ± 3.2, *p* = 0.7480, Dunnett's post hoc test) (Figures 1B(a,g),D and S1B(a,g,j,s),E) but was significantly reduced by Fsk (12.7 ± 3.1, *p* < 0.0001, Dunnett's post hoc test) (Figures 1B(d),D and S1B(d),E). By day 10, NESTIN levels declined across all treatments (Control: 26.1 ± 6.0, VPA: 3.5 ± 1.1, Rep: 16.7 ± 3.4, VPA + Rep: 12.7 ± 2.0, *p* < 0.0001, Dunnett's post hoc test) (Figure S1C,E), indicating reduced NC‐like progenitor across conditions.

Together, these results demonstrate that Fsk, Rep, and their combination significantly upregulated SOX10 expression while downregulating NESTIN in KC‐NCs (Figure 1C,D). Notably, Fsk and Rep treatments led to morphological changes, with cells adopting an elongated appearance compared to the smaller, spindle‐shaped morphology typical of KC‐NCs (Figure 1A(a–d)). Quantitative analysis of cell size (mean area per cell) revealed that treatment with Fsk, Rep, and their combination (Fsk + Rep) resulted in a mean cell area that was 1.5, 2.5, and 3 times larger, respectively, than the control (Control: 1106.9 ± 423.0 μm^2^; Fsk: 1678.8 ± 501.3, *p* = 0.0024; Rep: 2774.2 ± 916.3, *p* < 0.0001; Fsk + Rep: 3276.6 ± 1096.9, *p* < 0.0001) (Figure 1E).

To further characterize these morphological changes, we evaluated cell eccentricity (f), a measure of cell elongation. An f value approaching 1.0 indicates a highly elongated, bipolar morphology, while values closer to 0.0 suggest a circular shape (Figure 1F). Although Fsk‐treated cells did not exhibit significant elongation (*p* = 0.1522), Rep alone and the Fsk + Rep combination showed significantly increased elongation (*p* < 0.0001, Dunnett's post hoc test) compared to the control cells.

Moreover, KC‐NC yield was substantially reduced following Fsk and Rep treatment. Compared to the untreated control, Fsk‐treated cells showed a 32% yield (a threefold reduction), Rep‐treated cells yielded 17% (a sixfold reduction), and the Fsk + Rep combination yielded only 11% (a ninefold reduction) relative to control (100%) after 7 days of induction (Figure 1G). This reduction in cell yield further suggests that Fsk and Rep may have suppressed the proliferation of induced NC cells within KC cultures.

### 2.2 Chemical upregulation of SOX10 enhanced SC‐specific gene expression in KC‐NCs

Next, we conducted quantitative RT‐PCR to determine if small molecule‐induced upregulation of SOX10 impacted the expression of other genes related to NC identity and potential NC differentiation. Upon treatment with Forskolin (Fsk) and RepSox (Rep), we observed a substantial increase in *SOX10* mRNA levels on day 7 (Figure 1H): approximately 13‐fold with Fsk, 20‐fold with Rep, and nearly 30‐fold with their combined treatment (Fsk + Rep). However, other NC genes such as *FOXD3, PAX3, NESTIN, HNK1, TRKC, and TFAP2A* were significantly downregulated compared to FI‐induced cells (Figure 1H).

In contrast, genes associated with SC identity were upregulated in response to Fsk, Rep, and Fsk + Rep treatments (Figure 1I). Specifically, *S100B, PLP1*, and *MBP* mRNA levels increased by 20‐ to 40‐fold compared to controls, and Fsk also significantly elevated *KROX20, MPZ*, and *GFAP* expression. Both Fsk and Rep treatments upregulated *ERBB2* and *ERBB3* genes, which are critical for SC‐neuron communication through their interaction with NRG1 on neuronal axons.

We further examined genes associated with melanocytic, smooth muscle (SM), and neuronal lineages (Figure 1J). The melanocytic gene *PMEL* was highly upregulated (15‐ to 35‐fold) following Fsk and Rep treatments, though *MITF* showed no significant change. Rep alone significantly upregulated SM‐associated genes *ACTA2* (fourfold) and *CNN1* (10‐fold), while Fsk + Rep in combination further increased their expression (10‐ to 20‐fold). Interestingly, Fsk alone, but not Rep, somewhat upregulated neuronal genes *BRN2, ASCL1, TH*, and *NEUROD1* (twofold to fourfold).

### 2.3 Lentiviral expression of SOX10 drives SC gene expression in KC‐NC cells

**FIGURE 2:**
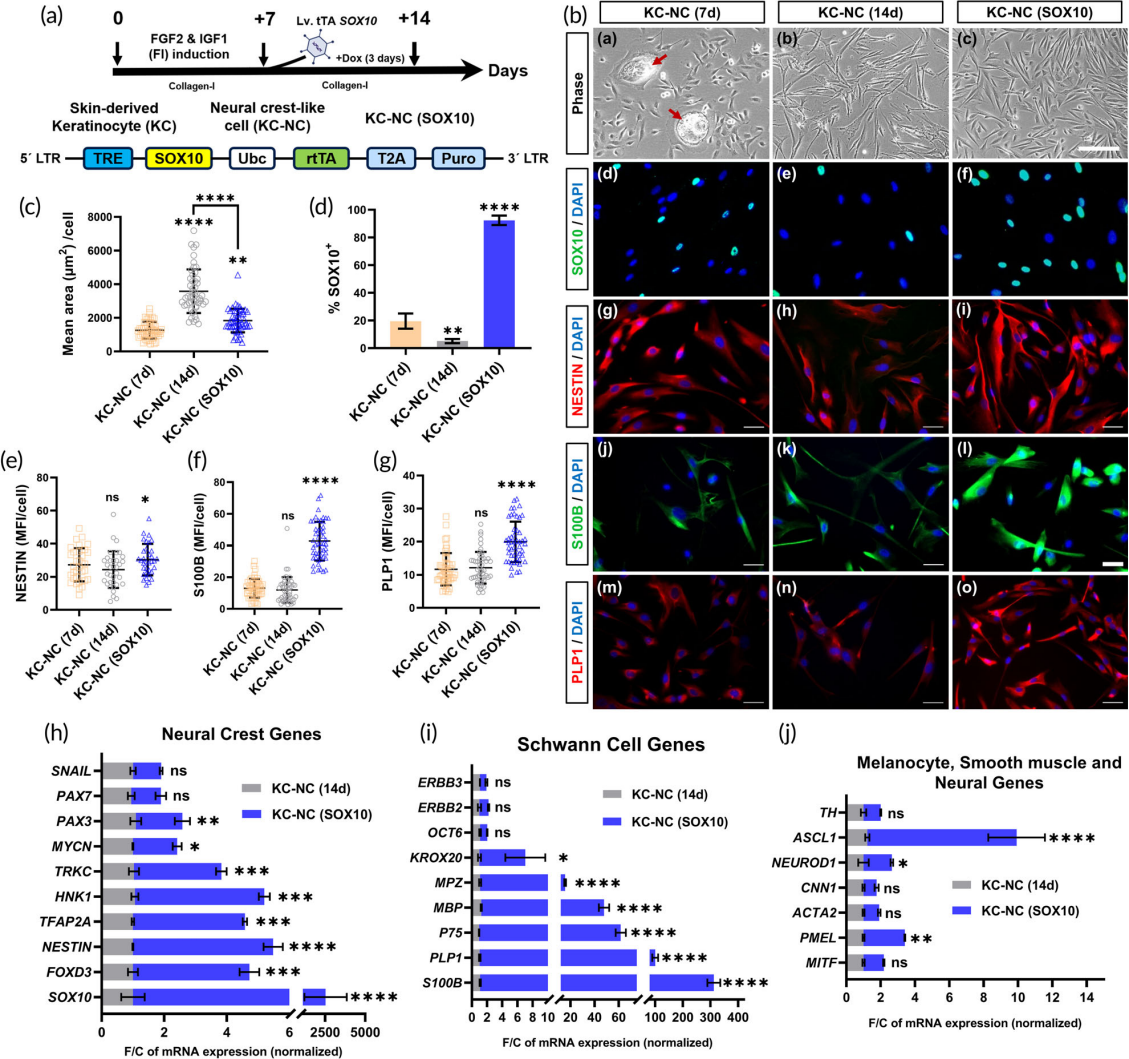
SOX10 expression upregulates Schwann genes in the KC‐NC cells. (A) Schematic representation of the reprogramming strategy to obtain KC‐NCs via FI induction for 7 days followed by overexpression of *SOX10* to generate the KC‐NC (SOX10) cells. Schematic of the lentiviral vector enabling doxycycline (Dox) inducible of *SOX10* gene expression. (B) Representative phase (a–c) and immunofluorescence images of (d–f) nuclear SOX10 (green), DAPI (blue), (g–i) cytoplasmic NESTIN (red), DAPI (blue), (j–l) cytoplasmic S100B (green), DAPI (blue), and (m–o) cytoplasmic PLP1 (red), DAPI (blue) for the NC‐like cells (KC‐NCs) after 7 days (7 days), 14 days (14 days), and genetic overexpression of *SOX10* for 3 days, respectively. Scale bar: a–c = 200 μm, d–l = 50 μm. (C) Quantification of average cell area, (D) Percentage (%) of SOX10^+^ cells, and (E–G) mean fluorescence intensity (MFI) of cytoplasmic NESTIN, S100B, and PLP1 in experimental conditions KC‐NC (7 days), KC‐NC (14 days), and KC‐NC (SOX10) compared to the control, (KC‐NC, 7 days). Data are mean ± SD, one‐way analysis of variance (ANOVA) with Tukey's multiple comparison test; ns: *p* ≥ 0.05, *: *p* < 0.05, **: *p* < 0.005, ***p (<0.0005), ****: *p* < 0.0001. (H–J) Quantification of gene expression via quantitative RT‐PCR for key genes specific to (H) NC cells, (I) SCs, (J) melanocytes, smooth muscle cells, and neurons. Data are presented as mean ± SD of fold change (F/C) in mRNA level normalized to untreated NC cells (KC‐NCs of Day 14) and internally normalized to *RPL32* cycle number. Two‐tailed unpaired *t*‐test between the two groups, *n* = 3 different donors; ns: *p* ≥ 0.05, *: *p* < 0.05, **: *p* < 0.005, ***: *p* < 0.0005, ****: *p* < 0.0001.

To further investigate whether SOX10 upregulation promotes KC‐NC differentiation to the SC lineage, we expressed the *SOX10* gene using lentivirus. We cloned *SOX10* into a doxycycline (Dox)‐inducible lentiviral vector and transduced the NC cells after 7 days of FGF2 and IGF1 (FI) induction. Following transduction and selection, *SOX10* expression was induced with doxycycline (Dox, 1 μg/mL) for 3 days, resulting in transgene‐expressing cells referred to as “KC‐NC (SOX10)” cells (Figure 2A).

Morphological analysis showed that while day 7 KC‐NCs exhibited a smaller, spindle‐shaped appearance (Figure 2B(a)), day 14 KC‐NCs were notably larger and exhibited more elongated morphology (Figure 2B(b)), reflecting a shift in cell identity over time. Quantitative cell area analysis confirmed this observation, with day 14 KC‐NCs showing a significantly greater mean cell area compared to day 7 KC‐NCs (7 days: 1267.9 ± 500.6 μm^2^; 14 days: 3528.9 ± 1256.9 μm^2^; *p* < 0.0001) (Figure 2C). Interestingly, KC‐NC (SOX10) cells appeared smaller than the untransduced KC‐NCs (14d) (Figure 2B(c); mean area: 1836.3 ± 706.0 μm^2^; *p* < 0.0001, Tukey's post hoc test), possibly indicating a SOX10‐driven cytoskeletal organization.

Immunofluorescence revealed that SOX10 expression, initially present in 19.5 ± 5.5% of KC‐NCs on day 7, declined to 5.1 ± 1.5% (*p* = 0.0088, Tukey's post hoc test) by day 14. However, lentiviral overexpression of SOX10 led to a substantial increase in SOX10^+^ cells (92.3 ± 3.4%) (Figure 2B(d–f),D). While NESTIN, a NC marker, remained similar in KC‐NC (7d) and KC‐NC cells, KC‐NC (SOX10) cells showed a slight increase in NESTIN expression as compared to day 7 KC‐NCs (*p* = 0.0446, Tukey's post hoc test) (Figure 2B(g–i),E). Furthermore, S100B and PLP1, two key SC markers, were significantly upregulated in the KC‐NC (SOX10) cells (S100B: 42.7 ± 12.2, *p* < 0.0001; PLP1: 19.9 ± 6.1, *p* < 0.0001) compared to untransduced NC cells on day 7 and 14 (S100B: 13.1 ± 5.9 (KC‐NC, 7 days), 12.0 ± 8.1 (KC‐NC, 14 days); PLP1: 11.7 ± 4.9 (KC‐NC, 7 days), 12.2 ± 4.8 (KC‐NC, 14 days)) (Figure 2B(j–o),F,G), highlighting a pronounced shift toward a SC‐like phenotype.

RT‐PCR analysis confirmed the enhanced expression of key NC genes in KC‐NC (SOX10) cells, specifically genes responsible for maintaining NC identity and multipotency (4‐ to fivefold: *FOXD3, NESTIN, TFAP2A, HNK1, TRKC*; 2.5‐fold: *MYCN*, *PAX3*) (Figure 2H). There was also a slight upregulation of pluripotency genes (sevenfold: *SOX2*; fourfold: *OCT4, NANOG*; twofold: *KLF4*) (Figure S2A) but no significant difference in epigenetic genes, except for *DNMT3A* (threefold) (Figure S2B).

Consistent with our hypothesis, SC‐related genes were markedly upregulated in the KC‐NC (SOX10) cells, including *S100B* (300‐fold), *PLP1* (100‐fold), *P75* (60‐fold), *MBP* (50‐fold), *MPZ* (20‐fold), and *KROX20* (sevenfold) (Figure 2I). These findings further support the notion that *SOX10* overexpression strongly drives SC lineage commitment. However, there was no significant change observed for *OCT6, ERBB2/3* in KC‐NC (SOX10) cells as compared to the KC‐NCs on day 14.

Unlike Fsk + Rep treatment, *SOX10* overexpression did not affect SM genes (*ACTA2 and CNN1*) and increased slightly the melanocyte‐related *PMEL* (2.5‐fold) but not *MITF*. It also increased neuronal lineage associated genes (*NEUROD1*: 1.5‐fold; *ASCL1*: 10‐fold), indicating a potential for neuronal differentiation (Figure 2J).

### 2.4 Continuous *SOX10* expression is required for efficient differentiation of the KC‐NC (SOX10) cells toward Schwann lineage

**FIGURE 3:**
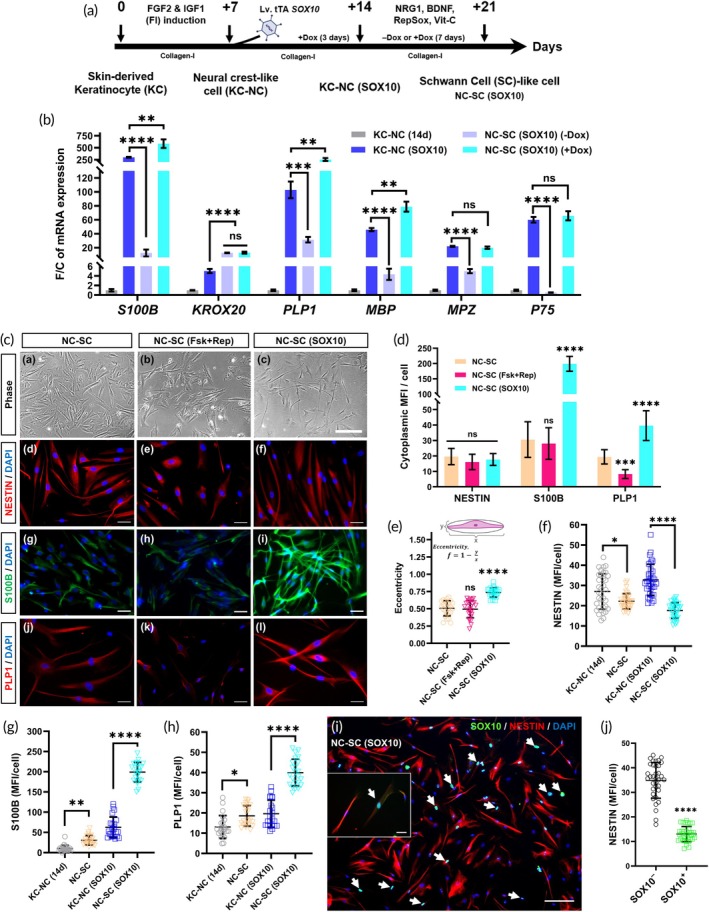
KC‐NC (SOX10) cells with continuous Dox‐induced SOX10 expression, differentiate into Schwann‐like cells more effectively than control or chemically (Fsk + Rep) treated KC‐NCs. (A) Schematic representation of obtaining KC‐NCs via 7 days of FI induction, followed by lentiviral transduction with a doxycycline‐regulatable SOX10 system to generate the KC‐NC (SOX10) cells by day 14. Then, the cells were differentiated toward Schwann lineage in presence or absence of doxycycline for 7 days to obtain SC‐like cells, NC‐SC (SOX10). (B) Quantification of gene expression via quantitative RT‐PCR for key genes specific to SCs. Data are mean ± SD, presented as fold change (F/C) in mRNA level normalized to control (untransduced KC‐NCs of Day 14) and internally normalized to *RPL32* cycle number, Two‐tailed unpaired *t*‐test between the respective two groups. (C) Representative phase contrast (a–c) and immunofluorescence images of the SC‐like cells stained for cytoplasmic (d–f) NESTIN (red) and DAPI (blue), (g–i) S100b (green) and DAPI (blue), and (j–l) Plp1 (red) and DAPI (blue). These cells were obtained from control, Fsk + Rep treated, and SOX10 expressed KC‐NCs respectively following a 7‐day SC differentiation protocol. Scale bar: a–c = 200 μm, and d–l = 50 μm. (d) Quantification of mean fluorescence intensity (MFI) of NESTIN, S100B, and PLP1 in the SC‐like cells after 7 days of differentiation. Data are mean ± SD, two‐way analysis of variance (ANOVA) with Tukey's multiple comparison test. (e) Analysis of bipolar morphology, typical for SC‐like cells, measured as eccentricity. (F–H) Quantification of MFI/cell for NESTIN, S100B, and PLP1 in the KC‐NCs and KC‐NC (SOX10) cells before and after SC differentiation. (I) SC‐like cells from KC‐NC (SOX10) co‐stained for nuclear SOX10 (green), cytoplasmic NESTIN (red), and nuclear DAPI (blue). Scale bar: 200 μm (inset at higher magnification = 20 μm). (J) Quantification of NESTIN (MFI) in the SC‐like cells after 7 days of differentiation. Here, the groups were defined based on SOX10 nuclear signal observed in the IF images: SOX10^+^ indicates strong nuclear localization; SOX10^−^ represents cells with undetectable or background‐level signal. Data are mean ± SD, one‐way analysis of variance (ANOVA) with Tukey's multiple comparison test, *n* = 3 different donors; ns: *p* ≥ 0.05, *: *p* < 0.05, **: *p* < 0.005, ***p: <0.0005, ****: *p* < 0.0001.

Real‐time PCR data showed that *SOX10*‐overexpressing KC‐NCs exhibit a strong tendency toward the Schwann lineage without SC‐specific differentiation medium. To investigate whether continuous *SOX10* expression was necessary for efficient SC differentiation, we coaxed KC‐NC (SOX10) cells to differentiate into Schwann‐like (NC‐SC) cells over 7 days, in the presence or absence of doxycycline (Dox) (Figure 3A). RT‐PCR analysis of the SC‐associated markers revealed that without Dox, differentiation led to significant downregulation of the key SC genes (*S100B, PLP1, MBP, MPZ*, and *P75*), whereas continuous Dox presence significantly enhanced their expression (Figure 3B). For example, the SC‐like cells obtained from the KC‐NC (SOX10) cells without Dox showed significantly reduced expression of *S100B* (12.5×), *PLP1* (3.3×), *MBP* (11.3×), *MPZ* (4×), and *P75* (30×), highlighting the necessity of sustained *SOX10* expression for effective SC differentiation. Interestingly, *KROX20* expression increased ~threefold post‐differentiation in both Dox‐treated and untreated groups, suggesting SOX10‐independent regulation. These findings confirm that sustained SOX10 expression is essential for efficient SC lineage commitment in the KC‐NC (SOX10) cells. Consequently, in all subsequent experiments, differentiation of these cells was consistently carried out in the presence of doxycycline in the culture media.

### 2.5 KC‐NC (SOX10) cells show superior SC‐like differentiation compared to untreated and chemically treated NCs

So far, we have observed that SOX10 upregulation in KC‐NCs either by chemical (Fsk + Rep) or genetic means significantly increased SC‐specific markers both at the mRNA and protein levels. Next, we evaluated the SC differentiation potential of the cells with elevated *SOX10* levels either by chemical or genetic means as compared to the untreated KC‐NC cells (Figure 3A,C,D). Morphologically, the NC‐SC (SOX10) cells displayed an elongated, bipolar shape, while NC‐SC and NC‐SC (Fsk + Rep) appeared less elongated (Figure 3C(a–c)). Quantitative analysis of cell eccentricity (Figure 3E) confirmed that NC‐SC (SOX10) cells were significantly more elongated (0.73 ± 0.06, *p* < 0.0005) than both control, NC‐SCs (0.52 ± 0.10) and NC‐SCs (Fsk + Rep) cells (0.49 ± 0.13). There was no significant difference (*p* = 0.901, Tukey's post hoc test) between the NC‐SC and NC‐SC (Fsk + Rep) group. SCs typically show long processes and display a high degree of eccentricity, indicating a strong orientation along one axis, which may help them to interact with and ensheathe neuronal axons in preparation for myelination.^22^, ^23^, ^24^, ^25^

NESTIN immunostaining (Figure 3C(d–f),D) revealed no significant differences among the three SC‐like cell groups as they all exited from their progenitor state. However, SC‐specific markers S100B and PLP1 were markedly elevated in NC‐SC (SOX10) cells, by sixfold and twofold, respectively, compared to control, NC‐SCs (*p* < 0.0001). While S100B levels did not differ (*p* = 0.6182) between the NC‐SC and NC‐SC (Fsk + Rep) groups, PLP1 was significantly downregulated (*p* = 0.002, Tukey's post hoc test) in the NC‐SC (Fsk + Rep) group, compared to the NC‐SC cells. Therefore, we proceeded with the genetically SOX10 upregulated cells, KC‐NC (SOX10) for the rest of the experiments.

The KC‐NC (SOX10) cells showed enhanced transition from progenitor to differentiated (SC‐like) state than control KC‐NCs. NESTIN expression (Figures 2B(h,i) and 3C(d,f),F) was markedly reduced in the differentiated cells, NC‐SC (SOX10) cells (17.7 ± 3.8, *p* < 0.0001), versus the KC‐NC (SOX10) cells (32.8 ± 7.7) unlike the control group (KC‐NC: 27.0 ± 8.7, NC‐SC: 22.2 ± 3.8, *p* = 0.0121, Tukey's post hoc test). Furthermore, NC‐SC (SOX10) cells showed substantial increases in S100B and PLP1 expression (Figures 2B(k,l) and 3C(j,l),G,H) compared to the control group (S100B: KC‐NC vs. NC‐SC, *p* = 0.0026; KC‐NC (SOX10) vs. NC‐SC (SOX10), *p* < 0.0001; PLP1: KC‐NC vs. NC‐SC, *p* = 0.0142; KC‐NC (SOX10) vs. NC‐SC (SOX10), *p* < 0.0001, Tukey's post hoc test), indicating their enhanced Schwann lineage commitment. This observation is further supported by immunostaining (Figure 3I), where NC‐SC (SOX10) cells exhibited nuclear SOX10 (green, shown by white arrow) with low cytoplasmic NESTIN (red) (13.0 ± 3.1), distinguishing them from the neighboring high‐NESTIN cells (34.9 ± 7.3, *p* < 0.0001) lacking SOX10 expression (Figure 3J).

Taken together, continuous SOX10 overexpression not only enhances SC gene expression during differentiation but also drives a more efficient transition from progenitor to SC‐like state, underscoring its role in SC lineage specification.

### 2.6 NRG1 immobilization and SOX10 overexpression synergistically enhance SC marker expression, transforming the KC‐NC cells into efficient SC‐like cells

Previously, we demonstrated that culturing NC cells on NRG1‐immobilized fibers^23^ promotes SC‐like morphology and upregulation of mature SC markers, via YAP1 nuclear localization and activation.^22^ Building on this, we hypothesized that differentiating SOX10‐overexpressing cells, KC‐NCs (SOX10) on an NRG1‐immobilized surface (iNRG1) would further potentiate their SC maturation through sustained NRG1‐ERBB2/3 signaling.

**FIGURE 4:**
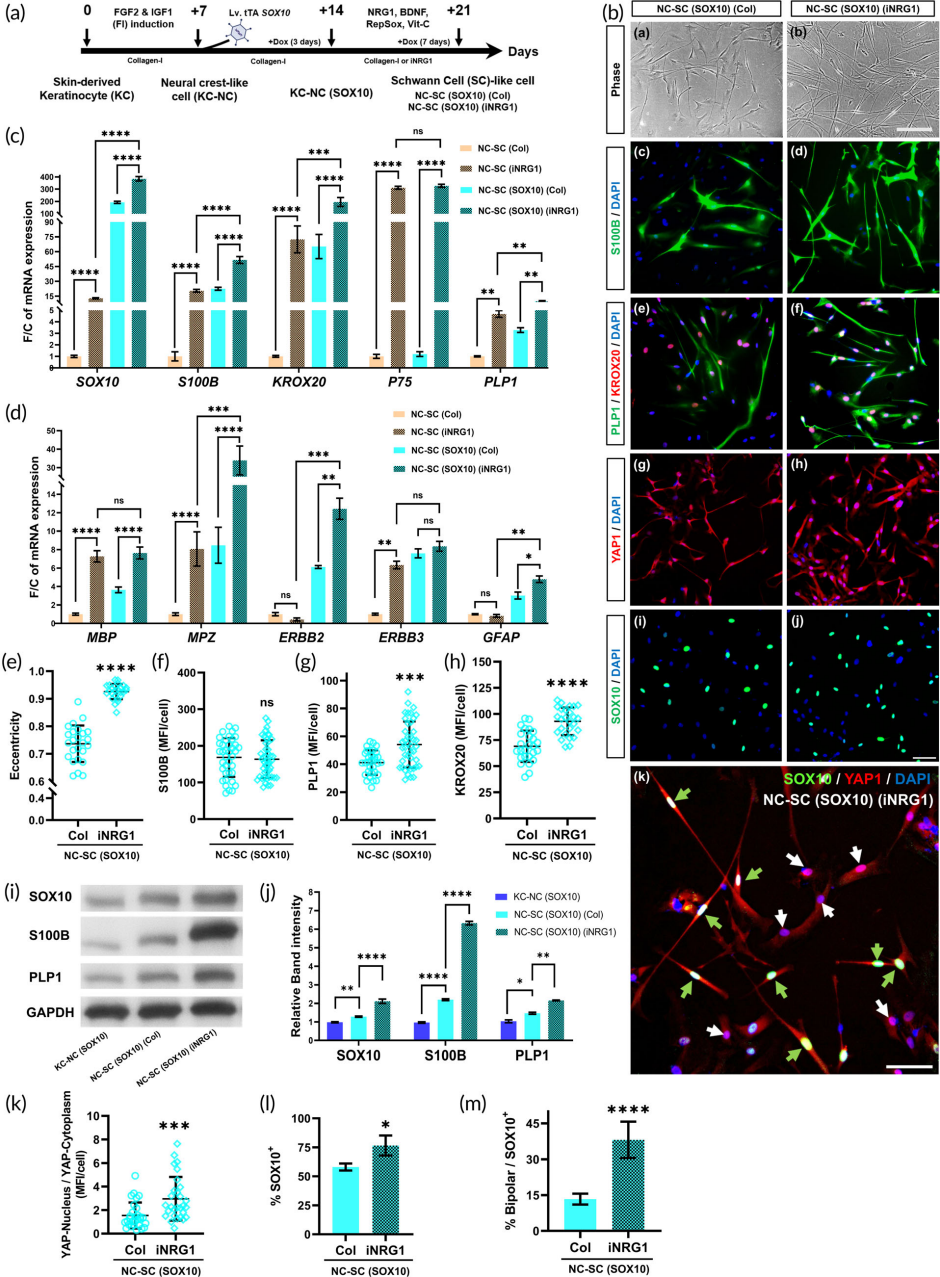
NRG1 immobilization and SOX10 expression synergistically enhance SC marker expression, promoting efficient SC differentiation of the KC‐NC cells: (A) Schematic representation of obtaining KC‐NCs via 7 days of FI induction, followed by lentiviral transduction with a doxycycline‐regulatable SOX10 system to generate the KC‐NC (SOX10) cells. Then, the cells were differentiated toward Schwann lineage in presence of doxycycline either on Collagen‐I (Col) or immobilized NRG1 (iNRG1) for 7 days to obtain SC‐like cells, NC‐SC (SOX10). (B) Representative (a, b) phase contrast and (c–i) immunofluorescence images of the SC‐like cells derived from KC‐NC (SOX10), differentiated on Col or iNRG1 surface, stained for (c, d) S100B (green) and DAPI (blue), (e, f) PLP1 (green) and KROX20 (red), and DAPI (blue), (g, h) YAP1 (red) and DAPI (blue), (i, j) SOX10 (green) and DAPI (blue) (k) SOX10 (green) and YAP1 (red), DAPI (blue), respectively. SOX10^+^ cells are marked with green arrows, while SOX10^−^ cells are marked with white arrows. Scale bar: a, b = 200 μm, c–i = 100 μm. (C, D) Quantification of SC‐specific gene expression by quantitative RT‐PCR, presented as fold change (F/C) in mRNA levels normalized to the SC‐like cells derived from control KC‐NCs on Col, with internal normalization to *RPL32* cycle count. Data are mean ± SD, one‐way ANOVA with Tukey's multiple comparisons test, *n* = 3 donors. (e) Quantification of cell eccentricity to evaluate the bipolar morphology of the cells typical for the SCs. (f–h) Quantification of mean fluorescence intensity (MFI) for cytoplasmic (f) S100B and (g) PLP1, and nuclear (h) KROX20. (i) Representative western blot detecting SOX10, S100B, PLP1, and GAPDH in KC‐NC (SOX10), and the SCs differentiated either on collagen or iNRG1 surface. (j) Quantification of the protein expression level, normalized to the KC‐NC (SOX10) on collagen. Data are mean ± SD, one‐way ANOVA with Dunnett's multiple comparisons test. (k) YAP1 (Nuclear/Cytoplasmic) analysis between the SC‐like cells obtained from KC‐NC (SOX10) cells either on Col or iNRG1. (l) Analysis of the percentage (%) of SOX10^+^ cells followed by the (M) Percentage of SOX10^+^ cells with bipolar morphology. Data are mean ± SD, Two‐tailed unpaired *t*‐test between the two groups; ns: *p* ≥ 0.05, *: *p* < 0.05, **: *p* < 0.005, ***p: <0.0005, ****: *p* < 0.0001.

To this end, control KC‐NC and KC‐NC (SOX10) cells were differentiated on either collagen or iNRG1 for 7 days, from day 14 to day 21 (Figure 4A). Quantitative RT‐PCR analysis showed a marked upregulation of SC‐specific genes (*p* < 0.0001: *SOX10, S100B, KROX20, P75, MBP, MPZ; p* < 0.005: *PLP1*, Tukey's post hoc test) on iNRG1 compared to collagen (Figure 4C,D), for both Control and SOX10 overexpressing KC‐NCs. On iNRG1, *SOX10* expression increased nearly 10‐fold in the SC‐like cells derived from control KC‐NCs, but 400‐fold in the *SOX10*‐expressing KC‐NCs. Notably, *S100B, KROX20, PLP1, MPZ*, *ERBB2*, and *GFAP* genes exhibited a similar pattern, with expression on iNRG1 surpassing levels observed on collagen for NC‐SC or NC‐SC (SOX10) cells, indicating synergistic enhancement of SC gene expression by SOX10 and iNRG1 signaling. On the other hand, *P75, MBP*, and *ERBB3* increased by iNRG1 but were not affected by SOX10 expression (Figure 4C,D).

In agreement, the SC‐like cells derived from KC‐NC (SOX10) cells on iNRG1 displayed highly elongated, bipolar morphology typical of myelinating SCs (Figure 4B(a,b)), with eccentricity values close to 1.0 (0.93 ± 0.03 on iNRG1 vs. 0.74 ± 0.07 on collagen, *p* < 0.0001, two‐tailed unpaired *t*‐test) (Figure 4E). Similarly, on iNRG1, mature SC markers such as S100B, PLP1, and KROX20 were upregulated in the SC‐like cells compared to SOX10‐overexpressing KC‐NC‐derived cells on collagen as shown by immunostaining (S100B: *p* = 0.8627, PLP1: *p* = 0.0001, KROX20: *p* < 0.0001, two‐tailed unpaired *t*‐test, Figure 4B(c–f),F–H) or western blot analysis (S100B: *p* < 0.0001, PLP1: *p* = 0.0056, Figure 4I,J)). These results indicate that iNRG1 increases mature SC protein expression and influences morphological changes (bipolar shape) in SC‐like cells derived from KC‐NC (SOX10) cells.

In line with iNRG1's role in promoting SC differentiation via YAP1 activation, we observed significantly increased nuclear YAP1 levels in the NC‐SC (SOX10) cells on iNRG1 compared to collagen (on Col: 1.5 ± 1.1, on iNRG1: 3.1 ± 2.2, *p* = 0.0008, Two‐tailed unpaired *t*‐test) (Figure 4B(g,h),K). Note that since YAP1 was also present in the cytoplasm of differentiated cells, the data were presented as YAP‐nucleus/YAP‐cytoplasm for comparison between the groups.

We have consistently observed the translocation of YAP1 from the cytoplasm to the nucleus during SC differentiation, as previously reported by our group.^22^ In undifferentiated KC‐NCs, both untransduced and those overexpressing SOX10, YAP1 remains localized predominantly in the cytoplasm (Figure S3A(a,b)). Upon differentiation (on collagen substrates), nuclear translocation of YAP1 was observed in the SCs derived from both control and SOX10‐expressing KC‐NCs (Figure S3A(c,d)).

The enhanced differentiation on iNRG1 was also found correlated with sustained *SOX10* expression as shown in Figure 4C. KC‐NC (SOX10)‐derived SC‐like cells on iNRG1 had a higher percentage of SOX10^+^ cells (76.6 ± 8.7% vs. 58.1 ± 3.0% on collagen, *p* = 0.0249, Two‐tailed unpaired *t*‐test) (Figure 4B(i,j),L). Furthermore, a higher proportion of these SOX10^+^ cells adopted bipolar morphology on iNRG1 (38.1 ± 7.6% vs. 13.4 ± 2.3% on collagen, *p* < 0.0001, two‐tailed unpaired *t*‐test) (Figure 4B(k),M). While a significant number of SOX10^+^ cells adopted bipolar morphology on iNRG1, not all did (Figure 4B(k)), suggesting that additional factors may influence such morphological changes. Overall, our results show that the combination of NRG1 immobilization with *SOX10* overexpression is a powerful strategy to generate mature SC‐like cells from skin‐derived KC‐NC.

## 3 DISCUSSION

Previously, we reported a simple and robust protocol utilizing a small‐molecule‐based approach to induce skin keratinocytes (KCs) to generate multipotent NC‐like cells (KC‐NCs) within 7 days from both neonatal and adult skin tissues.^10^, ^11^, ^26^ These KC‐NC cells were able to differentiate into traditional NC derivatives, such as melanocytes, neurons, SCs, and SM cells. Given the abundance and accessibility of human skin, human KC‐NC cells may provide large quantity of easily accessible and autologous source of multipotent stem cells for regenerative medicine, including cell therapy and disease modeling applications.

However, one major limitation of KC‐NC cells is the rapid loss of multipotency in culture, limiting their utility for regenerative applications. To overcome this issue, our laboratory previously reported the preservation of KC‐NC cell multipotency by treatment with WNT and BMP activators.^12^ Here, we explored an alternative approach by upregulating *SOX10*, a master regulator of NC cell formation, maintenance of multipotency, cell fate determination, and differentiation.^13^, ^14^ Interestingly, *SOX10* upregulation led to pronounced expression of SC‐specific genes (Figures 1I and 2I), suggesting a shift in lineage commitment. Both small molecule (Fsk + Rep) treatments, as well as lentiviral overexpression of *SOX10*, upregulated SC‐related genes, though Fsk + Rep treatments also led to elevated expression of melanocytic and SM markers (Figure 1J).

With Fsk + Rep treatment, expression of all NC‐associated genes, except *SOX10*, declined (Figure 1H). Instead, Fsk + Rep treatment induced significant upregulation of the melanocytic gene *PMEL*, possibly due to increased levels of SOX10, which is crucial for specification, maturation, and maintenance of SCs and melanocytes of NC origin.^27^ Interestingly, although SM differentiation has been reported to be suppressed by SOX10 expression^14^ and TGFβ inhibition,^28^ we observed increased expression of SM genes *ACTA2* and *CNN1* upon Fsk + Rep treatment (Fsk is an activator of cAMP, cyclic adenosine monophosphate and Rep is an inhibitor of ALK‐5 receptor of TGFβ signaling). However, neuronal genes were minimally affected by Fsk + Rep treatment (Figure 1J), in agreement with previous reports showing that neuronal differentiation requires SOX10 downregulation.^14^ On the other hand, genetic SOX10 overexpression induced both NC and SC markers with minimal impact on all other NC derivatives (Figure 2H), suggesting that Fsk + Rep treatment and SOX10 overexpression might induce distinct differentiation trajectories.

SCs are essential for myelinating peripheral neurons, and their activation following peripheral nerve injury highlights their therapeutic potential for nerve repair through SC transplantation or in vitro disease modeling.^29^ While most literature focuses on murine SCs, human SC studies remain scarce, and their differentiation processes are often lengthy and inefficient.^22^ For instance, Kim et al. (2014) reprogrammed human fibroblasts to multipotent NC‐like cells using SOX10 overexpression and WNT activation, which differentiated into neurons, SM cells, melanocytes, and glial cells.^30^ Mazzara et al. (2017) directly reprogrammed fibroblasts (human and murine) into SC‐like cells by overexpressing *SOX10* and *KROX20*, avoiding the pluripotency stage, though this approach yielded only 5% (human) to 12% (murine) SC‐like cells and required frequent cell sorting to separate the functional SCs.^31^ In contrast, our approach offers a robust 21‐day differentiation protocol (Figure 3A), including initial KC induction to NCs and subsequent SC differentiation via controlled expression of the *SOX10* gene only, and provides an efficient approach for generating human SCs. While our approach demonstrates improved efficiency, further in vitro and in vivo functional studies are required to validate and compare the effectiveness of these SCs with previously reported methods. To our knowledge, no studies have shown that SOX10 overexpression alone can drive human skin‐derived NC‐like cells specifically into the SCs lineage. Nonetheless, *SOX10* overexpression has also been utilized for generating oligodendrocytes, the myelin‐forming cells of the central nervous system. García‐León et al. generated O4^+^ oligodendrocytes from patient (Multiple Sclerosis and amyotrophic lateral sclerosis)‐derived human pluripotent stem cells (hPSCs) in 22 days. The process was conducted in two steps: (i) hPSC to NPC (10 days); and (ii) NPC to O4^+^ oligodendrocytes by continuous *SOX10* overexpression for 7–10 days.^32^

Notably, genetic SOX10 overexpression in KC‐NCs led to reduced cell size^33^ and increased NC‐associated gene expression (Figure 2C,H), suggesting potential dedifferentiation toward a more multipotent NC‐like state. SOX10 overexpression also upregulated S100B gene and protein levels, consistent with previous findings.^18^ Most notably, SOX10 expression was enhanced in the SC‐like cells, NC‐SC (SOX10), derived from the SOX10‐overexpressing KC‐NCs, differentiated on immobilized neuregulin 1 (iNRG1) coated surfaces (Figure 4C,L). Similarly, *KROX20* expression was significantly elevated in SOX10‐overexpressing cells on iNRG1‐ but not on collagen‐coated surface (Figure 4C,H), suggesting potential implication of the NRG1‐ERBB2/3 signaling pathway in NC maturation toward SCs. KROX20 is a transcription factor that is regulated by a network of transcription factors, including Oct6, Brn2, and Sox10 during SC differentiation^34^ and is critical for SC‐mediated peripheral myelination by controlling expression of mature myelination genes, such as *MBP* and *MPZ*.^35^

This observation prompted us to examine the transcriptional coactivators of the Hippo pathway, YAP and its paralog TAZ, known to regulate remyelination after peripheral nerve injury.^36^ Previously, we demonstrated that iNRG1 activates YAP/TAZ through nuclear relocation, promoting differentiation of the KC‐NCs into SCs capable of aligning with neuronal axons both in vitro and in vivo.^22^ Interestingly, SOX10 overexpression alone did not increase YAP or TAZ significantly (Figure S3B) compared to control SCs on collagen surface. On iNRG1, SOX10^+^ cells showed almost twofold increase both in nuclear YAP translocation (Figure 4B(g,h),K) and YAP/TAZ expression (Figure S3B). This finding suggests that YAP1 activation may be predominantly regulated by mechanotransducive cues associated with the iNRG1 substrate. This is consistent with in vivo observations, where SCs interact with axonal membrane‐bound NRG1 during myelination,^37^ a process known to influence mechanosensitive Hippo pathway via YAP/TAZ activation.^38^

Moreover, the combination of SOX10 expression on iNRG1 enhanced the fraction of SOX10^+^ cells with bipolar morphology (Figure 4B(k),M) typical of SCs, indicating a synergistic effect of these two signaling pathways on KC‐NC differentiation into mature SCs. In conclusion, our study demonstrates that SOX10 overexpression and NRG1 immobilization synergistically enhance SC lineage commitment and maturation of skin‐derived KC‐NC cells, enhancing their potential for use in regenerative medicine applications.

## 4 EXPERIMENTAL

### 4.1 Isolation of Keratinocytes (KC) from skin tissues

Keratinocytes (KCs) from neonatal skin tissues were obtained as described previously.^23^ Briefly, foreskin samples lacking hair follicles of 1 to 3‐day‐old neonates were obtained from the John R. Oishei Children's Hospital, Buffalo. The skin tissues were washed three times with phosphate‐buffered saline (PBS), chopped into small pieces (~3 × 1 mm), and enzymatically digested with dispase II protease (Sigma, St. Louis, MO) for 15–20 hr. at 4°C. Afterward, the epidermis was separated from the dermis using sterile, fine forceps. The separated epidermis layer was further digested with trypsin–EDTA (0.25%) (Life Technologies, Carlsbad, CA) for 10–15 min at 37°C and filtered through a 70 μm cell strainer (BD Biosciences, Franklin Lakes, NJ), and centrifuged at 300 × g and 4°C for 5 min. After removal of the supernatant, cell pellet was resuspended and plated on a confluent monolayer of growth‐arrested 3 T3/J2 mouse fibroblast feeder cells in keratinocyte growth medium (KCM) consisting of a 3:1 mixture of high glucose Dulbecco's Modified Eagle's Medium (DMEM, Catalog #11995‐040, Gibco, Grand Island, NY) and Ham's F‐12 medium (Life Technologies) supplemented with 10% (v/v) fetal bovine serum (FBS, Atlanta Biologicals, Flowery Branch, GA), 100 nM cholera toxin (Vibrio Cholerae, Type Inaba 569 B; Millipore, Burlington MA), 5 μg/mL transferrin (Life Technologies), 0.4 μg/mL hydrocortisone (Sigma), 0.13 U/mL insulin (Sigma), 1.4 × 10^−4^ M adenine (Sigma), 2 × 10^−9^ M triiodo‐L‐thyronine thyronine (Sigma), 1× antibiotic‐antimycotic (Life Technologies) and 10 ng/mL epidermal growth factor (EGF, BD Biosciences). Cells were cultured in KCM for 8–10 days or until a monolayer of keratinocytes was obtained. Then, the 3 T3/J2 feeder layer was detached by a 10 min versene (Life Technologies) treatment leaving the KC colonies on the plate. The remaining KC cells were treated with trypsin–EDTA (0.25%), followed by neutralization with a solution containing 10% FBS in PBS and plated in KC serum‐free growth medium (KSFM, Epilife medium with Human Keratinocyte Growth Supplement; Life Technologies). The cells were further expanded up to 1–3 passages before storage or induction to NC‐like cells.

### 4.2 Induction of KC into KC‐NCs

Tissue culture plates were coated with collagen (10 μg/mL collagen type I in sterile water, rat tail; BD Biosciences) for 3 h at 37°C followed by washing with sterile water once before cell seeding. KCs were seeded at a density of 5000–7000 cells/cm^2^ on collagen‐coated dishes in the presence of NC induction medium, comprising basal medium (EBM‐2 medium; Lonza, Catalog # CC4176, Basel, Switzerland) supplemented with 2% (v/v) FBS (Lonza), 10 μg per ml heparin (Lonza), 100 μg/mL ascorbic acid (Lonza), and 0.5 μg/mL hydrocortisone (Lonza), 1× Gentamicin/Amphotericin‐B (Lonza), 10 ng/mL fibroblast growth factor 2 (FGF2; BD Biosciences) and 10 ng/mL insulin‐like growth factor 1 (IGF1, Lonza). This medium was named FI medium. The medium was changed every alternate day. KC‐NCs were then separated from KC cultures between days 7 and 8 by short treatment with warm trypsin (1 min, 37°C), neutralized by 2% FBS in PBS, and centrifuged at 300 × g and 4°C for 5 min. The cells were then seeded on collagen I‐coated dishes for further expansion in FI medium.

For investigation of the effect of small reprogramming molecules (Forskolin, Fsk: 10 μM, Cayman Chemical, Catalog #14794; Valproic acid, VPA: 500 μM, Cayman Chemical, Catalog #13033; RepSox, Rep: 10 μM, Cayman Chemical, Catalog #14794), KCs were induced with the chemicals in the FI medium for 7 days or 10 days for 2 different time points. Untreated cells cultured in FI for 7 days served as controls.

### 4.3 Molecular cloning, viral packaging and infection

A tetracycline (tet)‐regulatable system was used to overexpress *SOX10* in KC‐NCs. To generate this vector, we first amplified SOX10 gene coding sequence (CDS) by PCR amplification of cDNA isolated from KC‐NCs after 6 days of NC induction. BamHI, EcoRI, PmeI, and BsrGI cloning sites were added at the ends of *SOX10* CDS. This was then cloned into the pNL‐EGFP/TREPittdU3 (Addgene) vector to generate pNL‐SOX10/TREPittdU3. To add rtTA and Puromycin sequences to this vector, we cloned it into the pNL‐TRE‐NANOG‐Ubc‐rtTa‐IRES‐Puro vector (previously published by our lab^39^) which was modified to include a PmeI and EcoRI cloning site flanking, eventually generating pNL‐TRE‐SOX10‐Ubc‐rtTa‐IRES‐Puro. Finally, IRES was replaced with the T2A peptide derived from the FUW‐OSKM vector (Addgene). The resulting vector pNL‐TRE‐Sox10‐Ubc‐rtTa‐T2A‐Puro was then expanded in STBL3 (Cat# 737303, Thermo Fisher Scientific) and plasmids were isolated using QIAGEN® Plasmid Plus Kit (Qiagen).

Complementary DNA (cDNA) for transcription factor (TF), SOX10, was cloned into a lentiviral Tet‐On system under the control of the tetracycline operator as presented in Figure 2A. The lentivirus packaging system consists of 3 plasmids: a plasmid containing the transgene (20 μg), pSPAX2 (20 μg), and pMD2G (5 μg), which were transfected into 293 T cells (at 60%–70% confluency in a P150 dish) using the standard calcium phosphate precipitation method.^40^ The medium was changed at 8 h post‐transfection and replaced with Optimem+Glutamax medium (Life Technologies, Catalog #51985‐034). The first batch of virus was harvested at 24 h post‐transfection, and the media was replaced by Optimem+Glutamax media supplemented with 5 mM sodium butyrate. The second batch of virus was harvested at 48 h post‐transfection. Both batches were pooled together and filtered through a 0.45‐μm filter (Millipore, Bedford, MA), pelleted by ultracentrifugation (50,000 g at 4°C for 2 h) and resuspended in fresh Optimem+Glutamax medium at 100× concentration as stock (pellet from 30 mL medium resuspended in 300 μL).

For lentiviral transduction, KC‐NCs were separated from KC cultures on day 7 and seeded on collagen‐I‐coated plate at 10,000 cells/cm^2^ in FI. A total of 24 h post‐seeding, the cells were infected by incubation with lenti‐*SOX10* (2 MOI, 4× conc. of stock, 4 μL diluted in 100 μL medium) and polybrene (8 μg/mL) in the FI medium. Fresh FI medium was added to the cells 8 hours after viral infection. 48 h post‐infection, the cells were selected by introducing puromycin (1 μg/mL) in the media and continued for 3 days. The cells were treated with doxycycline containing (2 μg/mL) medium for 3 days prior to analysis or passaging at day 14. The medium was changed every alternate day throughout the culture period.

### 4.4 Differentiation of the KC‐NCs toward SC fate

NCs of day 14 (control, chemically treated or lentivirus transduced) were either seeded on collagen‐coated tissue culture plate or NRG1 immobilized (iNRG1) non‐tissue culture plates at 10,000 cells/cm^2^. For iNRG1 coating, plates were incubated overnight at 4°C with NRG1‐Fc fusion protein (10 μg/mL, Sino Biological, Catalog # 11609‐H01H) in 1× PBS followed by PBS wash once before seeding the cells. SC differentiation was initiated by treating the cells with EBM2 basal medium supplemented with 1% FBS (Lonza), B27 supplement (1:100, Invitrogen), 1× Gentamicin (Lonza), 0.5× Glutamax (Invitrogen), Ascorbic acid (200 μg/mL), NRG1 (50 ng/mL, Sigma, Catalog # H7660), BDNF (10 ng/mL, Invitrogen, Catalog # PHC7074), RepSox (10 μM, Cayman Chemical, Catalog #14794) for 7 days. The medium was changed every alternate day throughout the culture period.

### 4.5 Quantitative real‐time PCR

Following the isolation of the KC‐NCs from KC colonies after small chemical treatment (Fsk, Rep, and combination), cells were seeded at 80% confluence (20,000 cells per cm^2^) on collagen‐I‐coated 6‐well plates. Untreated cells were used as control. For lentiviral transduction, KC‐NCs were seeded at lower density (10,000 cells per cm^2^). Both chemically treated and lentivirus transduced cells, along with untreated NC cells, were subsequently differentiated toward Schwann lineage on collagen‐ or iNRG1‐coated at a seeding density of 10,000 cells per cm^2^.

At the end of the experimental timelines (as shown in Figures 1A, 2A and 3A), total RNA was isolated using the RNeasy Mini Kit (Qiagen, Valencia, CA) following the manufacturer's protocol. cDNA was synthesized from 1 μg of isolated RNA per sample using the High‐Capacity cDNA Reverse Transcription Kit (Thermo Fisher Scientific, Catalog #4368814). To assess gene expression, real‐time PCR was performed in a CFX 96 real‐time system (Bio‐Rad, Hercules, CA) using PowerUp SYBR Green Master Mix (Thermo Fischer Scientific, Catalog # A25742) and the primer pairs listed in Table S1.

### 4.6 Immunocytochemistry (ICC)

Cells were washed with cold PBS (4°C) once and fixed for 10 min with 4% paraformaldehyde (Sigma), followed by permeabilization with 0.1% (v/v) Triton X‐100 (Sigma) in PBS for 10 min at RT. Next, samples were blocked using 5% (v/v) goat serum (Life Technologies) in PBS with 0.01% (v/v) Triton X‐100 for 1 h at room temperature. Immunostaining was performed using primary antibodies listed in Table S2, at 4°C overnight. Subsequently, cells were incubated with secondary antibodies: Alexa 488‐ or Alexa 594‐conjugated anti‐IgG antibody (1:400, Life Technologies) for 1 h at room temperature. Nuclei were stained using DAPI (4′,6‐Diamidino‐2‐Phenylindole) (Thermo Fisher Scientific, D1306). Cells stained with only secondary antibodies served as controls.

### 4.7 Western blot (WB)

Cell lysates were harvested from cell monolayers using a lab‐made standard lysis buffer [Composition: 100 μL (100×) cocktail protease inhibitor (Thermo Fisher Scientific), 3.33 mL (3×) blue loading buffer (Cell Signaling Technology, Danvers, MA, USA), 333 μL (30×) DTT (Cell Signaling Technology), and quantity sufficient water to make a total volume of 10 mL]. Lysates were denatured by incubation at 95°C for 5 min. The protein concentration was determined using the Bradford assay. Proteins were loaded at 20 μg per lane and were separated in precast 4%–20% Novex Tris–glycine gels (Thermo Fisher Scientific) by electrophoresis and subsequently transferred to nitrocellulose membranes (Millipore, Billerica, MA, USA) by the Trans‐Blot Turbo system (Bio‐Rad, Hercules, CA, USA). The membranes were blocked 5% (w/v) nonfat dry milk in Tris‐buffered saline with 0.1% Tween® 20 detergent (TBST) buffer (20 mM tris, 150 mM NaCl, and 0.1% Tween 20) for 1 h at room temperature. Subsequently, the membranes were incubated overnight at 4°C with primary antibodies (Table S2). Finally, the protein bands were visualized using horseradish peroxidase conjugated secondary antibodies and a chemiluminescence kit (Cell Signaling, Danvers, MA) according to the manufacturer's instructions. Luminescent blots were imaged using ChemiDoc™ Touch Imaging System (Bio‐Rad, Hercules, CA). Protein content was analyzed by densitometric analysis using the NIH image j software and normalized to GAPDH.

### 4.8 Fluorescence microscopy and image analysis

Immunostained images were obtained using a Zeiss Axio Observer Z1 inverted microscope (LSM 510; Zeiss, Oberkochen, Germany) equipped with an ORCA‐ER CCD camera (Hamamatsu, Japan). The images were captured using a fixed exposure time for each fluorescent dye for all the samples. Quantification of cell number and fluorescence intensity was measured using the NIH ImageJ software.

### 4.9 Statistical analysis

GraphPad Prism 8.0.2 software was used to perform statistical analysis. Two‐tailed Student's *t*‐test was used to compare two groups/time points. One‐way ANOVAs followed by Dunnett's/Tukey's post hoc test (as recommended) were used to compare more than two groups/time points. Statistical significance was set at a *p*‐value of <0.05 (*), *p* < 0.005 (**), *p* < 0.0005 (***), *p* < 0.0001 (****). Data were represented as mean ± SD for one representative experiment or multiple independent experiments. All experiments were repeated with at least three biological donors to ensure reproducibility.

## AUTHOR CONTRIBUTIONS

STA conceived the project and supervised the research. AKP and STA designed the experiments for the research and wrote the manuscript. PM and PL designed the plasmid for lentiviral overexpression experiments. AKP performed and analyzed all cell culture experiments, immunofluorescence staining, western blot, and imaging of the samples. PM performed preliminary cell culture experiments and relevant immunostaining with small molecules.

## CONFLICT OF INTEREST STATEMENT

All authors declared no competing interests.

## Supporting information


**Figure S1.** Evaluation of small molecule treatments to preserve NC‐like identity in the KC‐NCs. (A) Schematic representation of FGF‐2 and IGF1 (FI) mediated reprogramming of the skin‐derived KCs to NC‐like cells (KC‐NCs) in the presence of small chemicals such as forskolin (Fsk), valproic acid (VPA), RepSox (Rep), and their combinations. (B, C) Representative immunofluorescence images of the KC‐NCs for cytoplasmic NESTIN (red), nuclear SOX10 (green), and nuclear DAPI (blue) after 7 and 10 days of induction, respectively, with the chemicals; (a–c) Control, no small molecule treatment, (d–f) Fsk, (g–i) VPA, (j–l) Rep, (m–o) Fsk + VPA + Rep, (p–r) Fsk + VPA, (s–u) VPA + Rep, (v–x) Fsk + Rep. Scale bar: 200 μm. (D, E) Quantification of the percentage (%) of SOX10^+^ cells over the total number of KC‐NC cells and mean fluorescence intensity (MFI) of NESTIN (cytoplasmic) in control (FI) and treated cells evaluated after 7 and 10 days of induction. All data in bar graphs are presented as mean ± SD. All statistical tests were performed using One‐way analysis of variance (ANOVA) with Dunnett's multiple comparison test; ns: *p* ≥ 0.05, *: *p* < 0.05, **: *p* < 0.005, ***p: <0.0005, ****: *p* < 0.0001.

**Figure S2.** Evaluation of pluripotency and epigenetic markers in the KC‐NC (SOX10) cells. (A, B) Quantification of gene expression via quantitative RT‐PCR for key genes specific for controlling pluripotency and epigenetic regulation in KC‐NC (SOX10) cells compared to the KC‐NCs of Day 14. Data are presented as Mean ± SD of fold change (F/C) in mRNA level normalized to untransduced KC‐NCs (day 14) and internally normalized to *RPL32* cycle number. Two‐tailed unpaired *t*‐test between the two groups, *n* = 3 different donors; ns: *p* ≥ 0.05, *: *p* < 0.05, **: *p* < 0.005, ***: *p* < 0.0005.
**Figure S3.** SOX10 and YAP/TAZ expression in control and SOX10 overexpressing KC‐NCs: (A) Representative immunofluorescence images showing SOX10 (green) and YAP1 (red) in control and SOX10‐overexpressing cells on a collagen surface, both (a, b) before (day 14) and (c, d) after 1 week of Schwann differentiation (day 21). Scale bar: 100 μm. (B) Quantification of YAP and TAZ (key effectors of Hippo pathway) gene expression by quantitative RT‐PCR, presented as fold change (F/C) in mRNA levels normalized to the control KC‐NCs on Col, with internal normalization to *RPL32* cycle count. Data are Mean ± SD, one‐way ANOVA with Tukey's multiple comparisons test, *n* = 3 donors.

**Table S1.** List of primers.
**Table S2.** List of antibodies.

## Data Availability

All data needed to evaluate the conclusions in the paper are present in the paper and/or in the Supplementary Materials. Schematics were generated using Biorender (Biorender.com; publication and licensing rights agreement KN285K6M7S).
