# Supplementary material for: Enhanced Schwann cell differentiation of skin‐derived neural crest‐like stem cells through the synergistic action of SOX10 and immobilized NRG1 signaling
Source: Bioeng Transl Med. 2025 Aug 20;10(6):e70041. doi: 10.1002/btm2.70041 (PMC12617549; doi:10.1002/btm2.70041)
Supplement: Supplementary file 3 — Table S1. List of primers. Table S2. List of antibodies. [file BTM2-10-e70041-s003.docx]

**Supplementary Table S1: List of Primers**

| **Target Gene** | **Forward 5'→ 3'** | **Reverse 5'→ 3'** |
| --- | --- | --- |
| *SOX10* | CCTCACAGATCGCCTACACC | CATATAGGAGAAGGCCGAGTAGA |
| *PAX3* | AGCTCGGCGGTGTTTTTATCA | CTGCACAGGATCTTGGAGACG |
| *PAX7* | ACCCCTGCCTAACCACATC | GCGGCAAAGAATCTTGGAGAC |
| *SNAIL* | CCTCCCTGTCAGATGAGGAC | CCAGGCTGAGGTATTCCTTG |
| *MYCN* | CACGTCCGCTCAAGAGTGTC | GTTTCTGCGACGCTCACTGT |
| *HNK1* | CTCCTTCGAGAACTTGTCACC | GGGTCAGTGAAGCCCTTCTT |
| *TRKC* | ACGAGAGGGTGACAATGCTG | CCAGTGACTATCCAGTCCACA |
| *TFAP2A* | CTCCGCCATCCCTATTAACAAG | GACCCGGAACTGAACAGAAGA |
| *SOX2* | GCCGAGTGGAAACTTTTGTCG | GGCAGCGTGTACTTATCCTTCT |
| *OCT4* | GGGAGATTGATAACTGGTGTGTT | GTGTATATCCCAGGGTGATCCTC |
| *KLF4* | CAGCTTCACCTATCCGATCCG | GACTCCCTGCCATAGAGGAGG |
| *MYC* | CACGTCCGCTCAAGAGTGTC | GTTTCTGCGACGCTCACTGT |
| *NANOG* | CCCCAGCCTTTACTCTTCCTA | CCAGGTTGAATTGTTCCAGGTC |
| *HIF1Α* | GAACGTCGAAAAGAAAAGTCTCG | CCTTATCAAGATGCGAACTCACA |
| *ASCL1* | CCCAAGCAAGTCAAGCGACA | AAGCCGCTGAAGTTGAGCC |
| *TH* | GGAAGGCCGTGCTAAACCT | GGATTTTGGCTTCAAACGTCTC |
| *NEUROD1* | ATGACCAAATCGTACAGCGAG | GTTCATGGCTTCGAGGTCGT |
| *S100B* | TGGCCCTCATCGACGTTTTC | ATGTTCAAAGAACTCGTGGCA |
| *PLP1* | ACCTATGCCCTGACCGTTG | TGCTGGGGAAGGCAATAGACT |
| *MITF* | GCCTCCAAGCCTCCGATAAG | CATCTGCTCACGCATGAGTTG |
| *PMEL* | AGGTGCCTTTCTCCGTGAG | AGCTTCAGCCAGATAGCCACT |
| *ACTA2* | CTATGAGGGCTATGCCTTGCC | GCTCAGCAGTAGTAACGAAGGA |
| *CNN1* | GAACGTGGGAGTGAAGTACGC | CAGCCCAATGATGTTCCGC |
| *KDM6A* | TTCCTCGGAAGGTGCTATTCA | GAGGCTGGTTGCAGGATTCA |
| *KDM5D* | CAAGACCCGCTTGGCTACATT | TTGGACGCGAGGAGTAAATCT |
| *EZH1* | GTCACTGAACACAGTTGCATTG | TGCACAAAACCGTCTCATCTTC |
| *EZH2* | GGACCACAGTGTTACCAGCAT | GTGGGGTCTTTATCCGCTCAG |
| *HDAC1* | CTACTACGACGGGGATGTTGG | GAGTCATGCGGATTCGGTGAG |
| *HDAC2* | ATGGCGTACAGTCAAGGAGG | TGCGGATTCTATGAGGCTTCA |
| *DNMT3A* | CCGATGCTGGGGACAAGAAT | CCCGTCATCCACCAAGACAC |
| *DNMT3B* | AGGGAAGACTCGATCCTCGTC | GTGTGTAGCTTAGCAGACTGG |
| *KROX20* | TCAACATTGACATGACTGGAGAG | AGTGAAGGTCTGGTTTCTAGGT |
| *MPZ* | CATCGTGGTTTACACCGACAG | TGGAAGATCGAAATGGCATCTCT |
| *GFAP* | CTGCGGCTCGATCAACTCA | TCCAGCGACTCAATCTTCCTC |
| *OCT6* | CGCTCTACGGTAACGTGTTCT | CCAAGCCGGTGATCTCGTG |
| *ERBB2* | TGCAGGGAAACCTGGAACTC | ACAGGGGTGGTATTGTTCAGC |
| *ERBB3* | GGTGATGGGGAACCTTGAGAT | CTGTCACTTCTCGAATCCACTG |
| *MBP* | GGCCGGACCCAAGATGAAAA | CCCCAGCTAAATCTGCTCAGG |
| *FOXD3* | CGTTCAGCATCGAGAACATC | GAGAGTGGCACGCTAAGAAT |
| *NESTIN* | CAGCGTTGGAACAGAGGTTG | TGAGCGATCTGGCTCTGTAG |
| *P75* | CCTTACGGCTACTACCAGGATG | CACACGGTGTTCTGCTTGT |
| *RPL32* | GCCCAAGATCGTCAAAAAGAGA | TCCGCCAGTTACGCTTAATTT |
| *BRN2* | CGGCGGATCAAACTGGGATTT | TTGCGCTGCGATCTTGTCTAT |
| *YAP1* | TAGCCCTGCGTAGCCAGTTA | TCATGCTTAGTCCACTGTCTGT |
| *TAZ* | TCCCAGCCAAATCTCGTGATG | AGCGCATTGGGCATACTCAT |

**Supplementary Table S2: List of Antibodies**

| **Antibody** | **Catalog #** | **Company** | **Dilution for ICC (5%(v/v) goat serum in PBS)** | **Dilution for WB (5% milk in TBST buffer)** |
| --- | --- | --- | --- | --- |
| SOX10 | D5V9L | Cell Signaling | 1:200 | 1:1000 |
| KROX20 | NB100-92327 | Novus Biologicals | 1:400 | N/A |
| YAP1 | SC-101199 | Santa-Cruz | 1:200 | N/A |
| PLP1 | ab28486 | Abcam | 1:200 | 1:1000 |
| NESTIN | MAB5326 | Millipore Sigma | 1:200 | N/A |
| S100B | ab52642 | Abcam | 1:200 | 1:1000 |
| GAPDH | 2118  Clone #14C10 | Cell Signaling | N/A | 1:10,000 |
